# Supplementary material for: Preparation and Laboratory Testing of Polymeric Scale Inhibitor Colloidal Materials for Oilfield Mineral Scale Control
Source: Polymers (Basel). 2022 Oct 10;14(19):4240. doi: 10.3390/polym14194240 (PMC9572485; doi:10.3390/polym14194240)
Supplement: Supplementary file 1 [file polymers-14-04240-s001.zip › polymers-1912128-supplementary.pdf]

Supporting Information (SI) for

Synthesis and Laboratory Testing of Polymeric Scale Inhibitor Colloidal Materials for Oilfield Mineral Scale Control

Hanji Wang [1,2,†], Huaxia Dong [3,†], Xianbin Liu [1,2,*] and Ping Zhang [3,*]

1 School of Marine and Environmental Sciences, Tianjin University of Science and Technology (TUST), Tianjin 300457, China
2 Key Laboratory of Marine Resource Chemistry and Food Technology, Tianjin University of Science and Technology (TUST), Tianjin 300457, China
3 Department of Civil and Environmental Engineering, Faculty of Science and Technology, University of Macau, Macau, China* Correspondence: lxb0688@tust.edu.cn (X.L.); pzhang@um.edu.mo (P.Z.);
Tel.: +86-22-6060-2992 (X.L.); +853-8822-4917 (P.Z.)
† These authors contributed equally to this work.

1. Molecular structure of PSS inhibitor

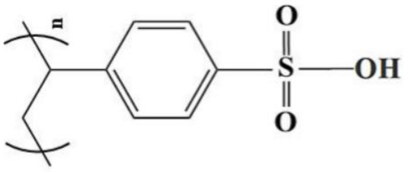

Figure S1. Molecular structure of PSS inhibitor.

2. Detailed procedure of testing BaPCI inhibition performance by inhibiting calcium phosphate and calcite scales

As for the test of inhibiting calcium phosphate scale, inside a volumetric flask of 500 mL, add BaPCI, $CaCl_2$ and, K_2HPO_4, and sodium tetraborate ($Na_2[B_4O_5(OH)_4]$) so that the Ca concentration is 100 mg L^{-1}, PO_4^{3-} concentration is 5 mg L^{-1}. Place this volumetric flask in a water bath of 80°C for a duration of 10 h. By the end of the 10 h, filter the mixture solution using filter paper. Then use ammonium molybdate spectrophotometry to measure the total phosphorus content in water and calculate the inhibition performance efficiency (η, %) following the below equation:

$$\eta = \frac{\rho_4 - \rho_3}{\rho_0 - \rho_3} \tag{1}$$

where ρ_4 (mg mL^{-1}) represents phosphate concentration after the completion of the tests with scale inhibitor added. ρ_3 (mg mL^{-1}) denotes phosphate concentration after the completion of the tests with no scale inhibitor added. ρ_0 (mg mL^{-1}) corresponds to phosphate concentration prior to conducting the test. Run similar tests with an initial Ca concentration of 200 mg L^{-1} and 400 mg L^{-1}, respectively.

As for the test of inhibiting calcite scale, inside a volumetric flask of 500 mL, add BaPCI, $CaCl_2$ and, $NaHCO_3$, and 20 mL borax buffer solution (3.8 g L^{-1}) so that Ca concentration is 240 mg L^{-1}, HCO_3^- concentration is 366 mg L^{-1}. Place this volumetric flask in a water bath at 80°C for a duration of 10 h. By the end of the 10 h, filter the mixture solution using filter paper. Once the solution is air cooled, transfer 25 mL filtered solution into a 250 mL Erlenmeyer flask. Add 5 mL KOH solution (200 g L^{-1}) and 0.1 g calcium-carboxylic acid indicator. Titrate the mixture solution inside the Erlenmeyer flask with EDTA standard solution until the solution color changes from purple red to bright blue. Determine the calcium concentration in solution and calculate the inhibition performance efficiency (η, %) following the below equation:

$$\eta = \frac{\rho_4 - \rho_3}{\rho_0 - \rho_3} \quad (2)$$

where ρ_4 (mg mL^{-1}) represents calcium concentration after the completion of the tests with scale inhibitor added. ρ_3 (mg mL^{-1}) denotes calcium concentration after the completion of the tests with no scale inhibitor added. ρ_0 (mg mL^{-1}) is actual Ca concentration prior to conducting the test. Run similar tests with an initial Ca concentration of 480 mg L^{-1} and 720 mg L^{-1}, respectively.

3. Detailed procedure of testing BaPCI inhibition efficacy by inhibiting barite scale

Induction time of barium sulfate ($BaSO_4$) nucleation with and without BaPCI materials was measured via nucleation kinetics (turbidity) tests. The experimental procedure is similar to that of He et al. [1] and Xiao et al. [2]. Aqueous solutions of $BaCl_2$ (0.6 mM) and Na_2SO_4 (0.6 mM) were prepared. These two solutions were then filtered with 0.2 mm filters to remove any fine particles suspended in solutions and stored inside an oven (25°C). No buffer was added to these solutions to avoid interference of buffer species to nucleation. The turbidity measurement setup comprises of a turbidity cell, water bath for temperature control, a turbidity meter (TL2300, HACH Co.) for turbidity measurement and a digital multimeter connected to a computer to record measurement results. A blank nucleation reaction was initiated by quickly adding the same volumes of $BaCl_2$ and Na_2SO_4 solutions into the turbidity cell and mixing with constant magnetic stirring. The temperature was controlled by the water bath. The measured turbidity was recorded through the duration of the nucleation kinetics experiment. In a separate test, a desired volume of BaPCI suspension was added drop wise into the prepared Na_2SO_4 solution (0.6 mM) under constant stirring. Subsequently, this inhibitor-containing Na_2SO_4 solution was mixed with $BaCl_2$ solution in 1:1 v/v in the turbidity cell for turbidity measurement, similar to the blank test.

4. Schematic of column setup for BaPCI column transport experiment

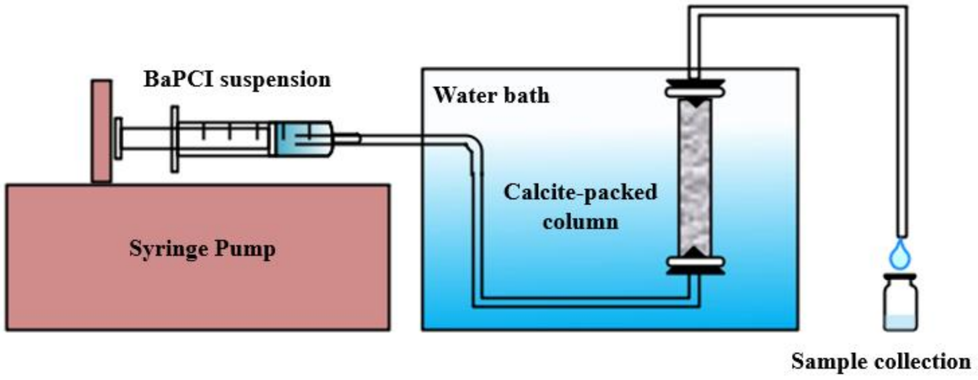

Figure S2. Schematic of column setup for the transport experiment.

5. Schematic of laboratory squeeze simulation experimental procedure

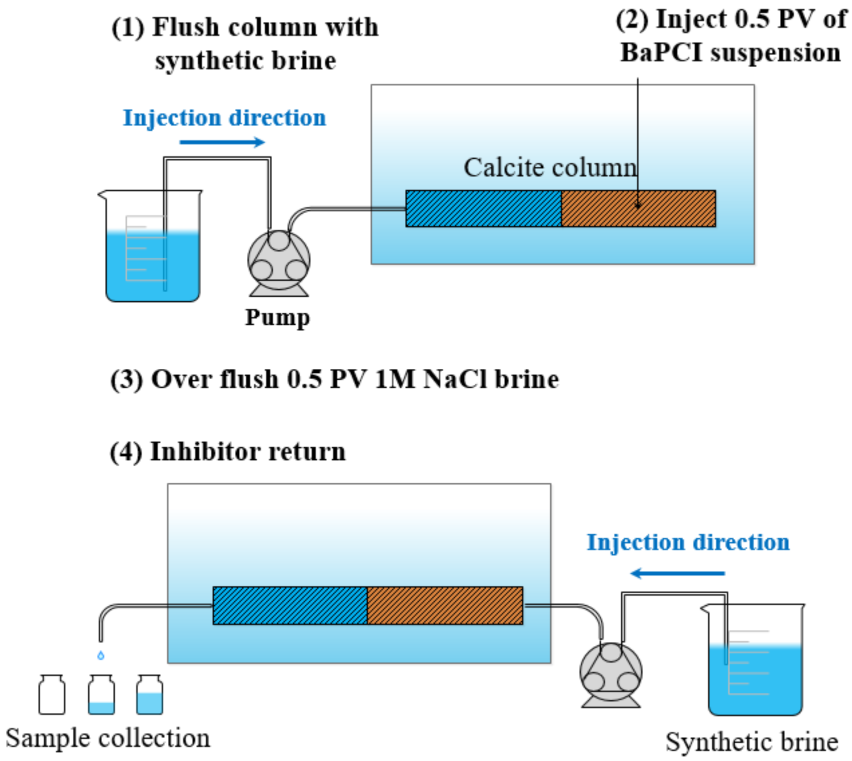

Figure S3. Illustration of laboratory squeeze simulation experimental procedures.

6. Understanding the BaPCI transport via advection-dispersive mechanism

6.1. Theory

From the standpoint of advection and diffusion, the transport of colloidal inhibitor materials can be mathematically described by one-dimensional advection-dispersive equation [3-5]:

$$R\frac{\partial C}{\partial t} - D\frac{\partial^2 C}{\partial x^2} + v\frac{\partial C}{\partial x} + J_d C = 0 \quad \text{(Eq. ESD-1)} \qquad (3)$$

C (mg L^{-1}) denotes the effluent BaPCI concentration at a given time; t (min) is the time; D (cm^2 min^{-1}) represents the hydrodynamic dispersion coefficient; x (cm) accounts for the distance of BaPCI transport which is the length of the column; and v (cm min^{-1}) is the linear pore velocity calculated as $v=Q/\pi r^2\varepsilon$, where Q is the flow rate (mL min^{-1}); ε is the calcite medium porosity, and r (cm) is the cross sectional radius of the column. Linear pore velocity is the average travel velocity for BaPCI particles in calcite medium. Two key parameters of critical importance in colloidal transport include R (unit less), the retardation factor, and J_d (min^{-1}), the first order deposition coefficient of BaPCI material to the surfaces of calcite medium. R characterizes the sorptive behavior of BaPCI to calcite surfaces due to retardation effect. J_d accounts for the deposition kinetics of BaPCI to calcite surfaces and is principally influenced by the energy barrier based upon the DLVO theory. The first term of Eq. ESI-1 represents the BaPCI concentration change at a given time; the second term characterizes the change in concentration due to diffusion/dispersion; the third term represents BaPCI concentration change associated with advection and the last term is the BaPCI mass removal modeled as a first order deposition process [3-5]. The mathematical solution to Eq. ESI-1 can be expressed as [3-5]:

$$C(x,t) = \frac{1}{2}\exp[\frac{(v-w)x}{2D}]\mathrm{erfc}[\frac{Rx-wt}{2(DRt)^{0.5}}] + \frac{1}{2}\exp[\frac{(v+w)x}{D}]\mathrm{erfc}[\frac{Rx+wt}{2(DRt)^{0.5}}] \quad \text{(Eq. ESD-2)} \qquad (4)$$

$$\text{and} \quad w = (v^2 + 4J_d D)^{0.5}$$

Thus, based on Eq. ESI-2, the breakthrough efficiency level (C/C_o) of the BaPCI materials from the column transport experiment can be mathematically calculated and compared with the experimentally obtained breakthrough level. The values of R and J_d in each transport study were acquired by minimizing the differences between the calculated effluent concentrations based on Eq. ESI-2 and the experimentally observed effluent concentrations via the least square method. Excel Solver function was used to find the R and J_d values.

6.2. Characterization of calcite formation medium via tracer test

A KBr tracer test was carried out to measure the PV and the hydrodynamic dispersion coefficient (D) of the packed column. According to the breakthrough curves of the tracer in each medium, the D values for each medium can be obtained by via Eq. ESD-2 by setting R to one and J_d to zero:

$$\frac{\partial C}{\partial t} = D\frac{\partial^2 C}{\partial x^2} - v\frac{\partial C}{\partial x} \qquad \text{(Eq. ESD-3)} \qquad (5)$$

where C (mg L^{-1}) is the effluent tracer concentration at a certain time and t (min) denotes the time. The tracer test was conducted at a flow rate of 6.5 mL hr^{-1}, corresponding to a pore velocity of 5.5 m d^{-1} and the measured D value is 0.017 cm^2 min^{-1}. D value (cm^2 min^{-1}) can be calculated as:

$$D = \alpha_d \times v \qquad \text{(Eq. ESD-4)} \qquad (6)$$

where α_d (cm) is the dispersivity and v (cm min^{-1}) is the linear pore velocity introduced above. Based on Eq. ESD-4, α_d for calcite medium at 5.5 m d^{-1} pore velocity is calculated to be 0.045 cm. α_d is a characteristic property of a formation media [5]. Thus, the dispersivity of calcite should be maintained the same for different pore velocities. The D values for other flow velocities can be easily calculated as the product of the dispersivity with the pore velocity, as shown in Table 2 of the main article.

7. Plot of the return curves on a linear scale of inhibitor concertation

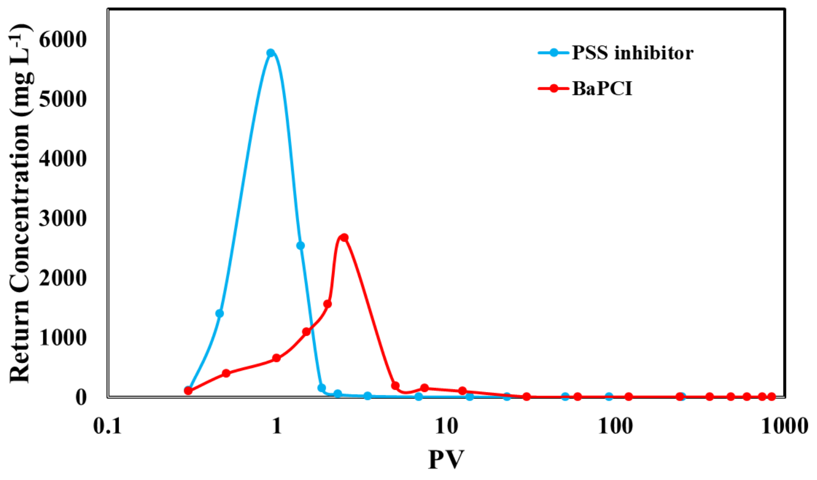

Figure S4. Inhibitor return curves plotted on a linear scale of inhibitor concentration.

8. Cumulative mass of inhibitor returned during the course of laboratory squeeze simulation experiments

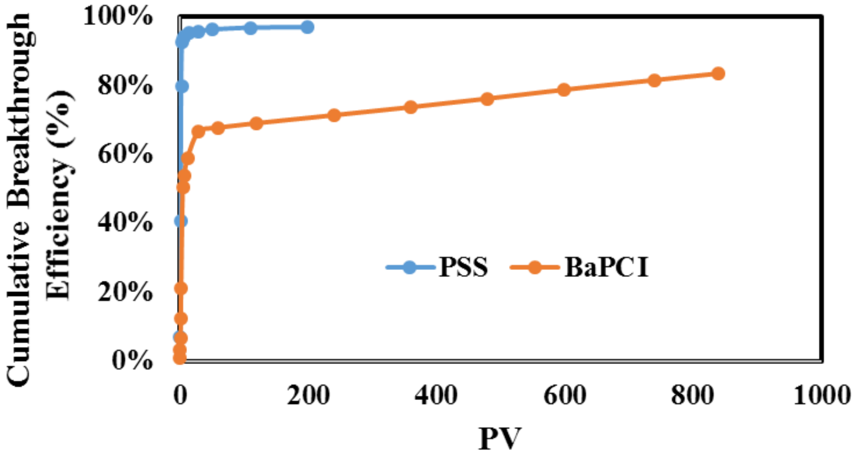

Figure S5. Cumulative mass of inhibitor returned during the course of laboratory squeeze simulation experiments.

References

1. He, S.; Kan, A.T.; Tomson, M.B. Mathematical inhibitor model for barium sulfate scale control. *Langmuir* **1996**, *12*, 1901–1905.
2. Xiao, J.; Kan A.T.; Tomson, M.B. Prediction of $BaSO_4$ precipitation in the presence and absence of a polymeric. *Langmuir* **2001**, *17*, 4668–4673.
3. Charbeneau, R.J. Groundwater hydraulics and pollutant transport, 1st ed.; Waveland Press: Long Grove, IL, USA, 2006.
4. Clark, M.M. Transport modeling for environmental engineers and scientists, 2nd ed.; John Wiley & Sons: Hoboken, NJ, USA, 2009.
5. Ryan, J.N.; Elimelech, M. Colloid mobilization and transport in groundwater. *Colloids Surf. A Physicochem. Eng. Asp.* **1996**, *107*, 1–56.
